# Research on Field Source Characteristics of Leakage Current of Arrester Based on TMR Sensor

**DOI:** 10.3390/s23083830

**Published:** 2023-04-08

**Authors:** Yameng Fu, Tanxiao Li, Yongfu Li, Xiaoxu Hu, Xiping Jiang, Yiran Dong, Pengcheng Zhao, Chuanxiang Yu, Jingang Wang

**Affiliations:** 1 State Key Laboratory of Power Transmission Equipment and System Security and New Technology, Chongqing University, Chongqing 400044, China; 2 Chongqing Electric Power Research Institute, Chongqing Electric Power Company, Chongqing 401123, China

**Keywords:** arrester, finite element method, TMR current sensor, magnetic field distribution

## Abstract

The status of zinc oxide (ZnO) arresters is directly related to the safety of power grids. However, as the service life of ZnO arresters increases, their insulation performance may decrease due to factors such as operating voltage and humidity, which can be identified through the measurement of leakage current. Tunnel magnetoresistance (TMR) sensors with high sensitivity, good temperature stability, and small size are excellent for measuring leakage current. This paper constructs a simulation model of the arrester and investigates the deployment of the TMR current sensor and the size of the magnetic concentrating ring. The arrester’s leakage current magnetic field distribution under different operating conditions is simulated. The simulation model can aid in optimizing the detection of leakage current in arresters using TMR current sensors, and the findings serve as a basis for monitoring the condition of arresters and improving the installation of current sensors. The TMR current sensor design offers potential advantages such as high accuracy, miniaturization, and ease of distributed application measurement, making it suitable for large-scale use. Finally, the validity of the simulations and conclusions is verified through experiments.

## 1. Introduction

Leakage current is the presentation form of the comprehensive effect of complex operating conditions on power equipment, which can represent the key information of the operation status and is convenient for online real-time monitoring [1,2,3]. Leakage current includes resistive current and capacitive current [4,5], with the resistive current only accounting for about 10% of the total current. Although the total leakage current does not obviously increase, the resistive component increases a lot when the equipment fails in the early stages. Therefore, many studies focus on the resistive component [6]. The measurement of leakage current is much more difficult than that of conventional current, and it is necessary to transform the weak electrical signal to measure it accurately.

Existing current measurement devices include conventional current transformers, fluxgate sensors, Hall current sensors, and fiber optic current transformers. Traditional current transformers are large, can only measure low- and power-frequency AC signals, and have low measurement accuracy. The fluxgate current sensor has a large size and large power consumption, so its development is limited. The non-contact leakage current sensor mainly adopts the Hall current sensor [7], but the low sensitivity and temperature sensitivity of the Hall current sensor limit its application in power systems. It cannot meet the current measurement technical requirements of digital and high-reliability online monitoring systems in the smart grid. Fiber optic current sensors are large and costly, making them unsuitable for large-scale use. Considering factors such as size, power consumption, accuracy, and cost, TMR sensors provide higher sensitivity and accuracy in detecting small currents [8,9]. In addition, the advantages of small size, low power consumption, high magnetic field resolution, and good temperature stability make TMR current sensors very suitable for weak magnetic field detection. Through the measurement of the magnetic field, the current at μA level can be measured accurately, so it is very beneficial for leakage current detection. For distributed measurement and data acquisition of power systems, TMR current sensors have the advantages of a simple structure and easy large-scale popularization. Therefore, TMR current sensors are selected to measure the leakage current.

Transmission and distribution lines are often subjected to lightning [10]. In the existing lightning protection devices in power systems, ZnO arresters are widely used because of their good nonlinear characteristics and excellent current capacity [11,12,13]. However, the arresters operate in a natural environment, so it is very easy to be exposed to moisture [14], reducing external insulation strength. When the dampness is serious, it may lead to the explosion of arresters, which poses a threat to the security of power networks. Therefore, it is of great significance to accurately diagnose the overvoltage and damp defects of arresters. The magnitude of the leakage current can reflect the operation state of the arrester [3,15]. The health state of the arrester is generally judged by the change of electrical parameters, such as leakage current caused by moisture [16]. Sayantan Das et al. explained the influence of the internal water content of arresters on their working characteristics [17]. Arup Kumar Das et al. proposed quantitatively estimating the intrusive water content in metal oxide arresters using the dielectric modulus technique [18]. Shenghui Wang et al. obtained the change in the electrical performance of the arrester by simulation of the damp test and obtained the temperature distribution of the arrester with an infrared imager [19].

At present, most of the research on the leakage current of arresters is focused on the measurement of current in a laboratory setting, and the current transformer is used to measure the leakage current for operating an arrester. There is a lack of related research using TMR current sensors to measure the leakage current. Simulation analysis is critical for large-scale high-voltage electrical equipment since the test analysis is time-consuming, some destructive tests are costly, and the test conditions are strict. In terms of simulation calculations, the electric field distribution [20,21] and temperature field distribution [22,23,24] of arresters are mainly studied. However, there are few achievements in the distribution of magnetic fields. As magnetic field sensors measure by converting magnetic fields into electrical signals, the magnetic field distribution of the arrester is of great significance for the research of leakage current measurement using a TMR current sensor. To intuitively show the leakage current and its field source distribution characteristics of the arrester under different operating conditions, this paper establishes a complete three-dimensional model of the ZnO arrester and measurement structure of the TMR sensor, determines the optimal size of the magnetic ring structure by parametric scanning, and analyzes the field source characteristics of the arrester under normal conditions through simulation to guide the installation and deployment of the sensor. Then, the leakage current characteristics under different AC voltages and the field source characteristics under different moisture conditions are studied systematically, and the magnetic field distribution characteristics of the arrester under different voltages and different moisture conditions are given. Finally, the effectiveness of the simulation model is verified by the leakage current experiment.

This paper fills the gap in the magnetic field simulation of the arrester. Simulation analysis of the magnetic field around the grounding wire of the arrester guides the installation and deployment of TMR sensors. The proposed TMR current sensor reduces the size and weight of the current sensor, making it more compact and easier to install. The magnetic concentrating ring not only increases the strength of the front-end induction signal, thereby increasing the sensitivity and accuracy of the current sensor but also reduces the influence of external magnetic fields, improving the anti-interference ability of the current sensor. The combination of simulation and experimentation provides a reference for judging the operating status of the arrester, which is beneficial for improving the operation and maintenance level of the arrester.

## 2. Design of TMR Leakage Current Sensor

### 2.1. Magnetic Field Measurement Principle of TMR Sensor

**Figure 1:**
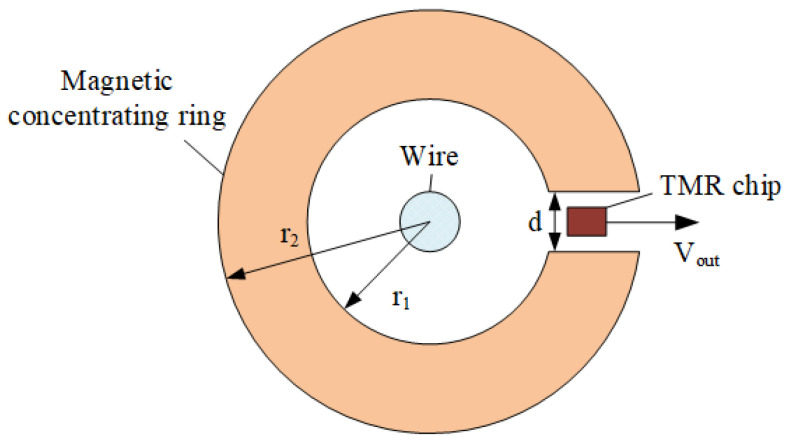
TMR current sensor structure.

The TMR sensor measures the leakage current by sensing the magnetic field signal and converting it into an electrical signal. Since the leakage current magnetic field is very weak, it is necessary to rely on the magnetic field amplification of the magnetic concentrating ring. The main circuit of the sensor is installed at the air gap of the magnetic ring to increase the magnetic induction intensity measured at the front end of the leakage current sensor. The current measurement of the sensor is shown in Figure 1.

According to the Ampere loop theorem:

(1) $$\oint H\cdot \mathrm{dl}=H_{C}\cdot \left(2\pi r_{0}-d\right)+H_{A}\cdot d=I$$

where H_C_ represents the magnetic field strength in the magnetic concentrating ring, H_A_ represents the magnetic field strength in the air gap, and r_0_ represents the average radius of the magnetic concentrating ring.

Substituting $B=\mu_{0}H_{A}=\mu H_{C}$ into (1):

(2) $$\frac{B\left(2\pi r_{0}-d\right)}{\mu }+\frac{Bd}{\mu_{0}}=I$$

The solution is as follows:

(3) $$B=\frac{\mu \mu_{0}}{2\pi r_{0}\mu_{0}+\left(\mu -\mu_{0}\right)d}I$$

Since the magnetic permeability of magnetic concentrating ring material is much greater than that of air, (3) can be simplified to:

(4) $$B=\frac{\mu_{0}}{d}I$$

When no magnetic ring is used, the magnetic induction intensity B_0_ at the center of the air gap is:

(5) $$B_{0}=\frac{\mu_{0}I}{\pi (r_{1}+r_{2})}$$

The ratio of magnetic induction intensity at the center of the air gap after and before using the magnetic concentrating ring is:

(6) $$\frac{B}{B_{0}}=\frac{\pi (r_{1}+r_{2})}{d}$$

It can be seen from (6) that the air gap width d should be as small as possible to obtain the maximum magnetic induction intensity.

### 2.2. Design of Magnetic Concentrating Ring Structure

**Figure 2:**
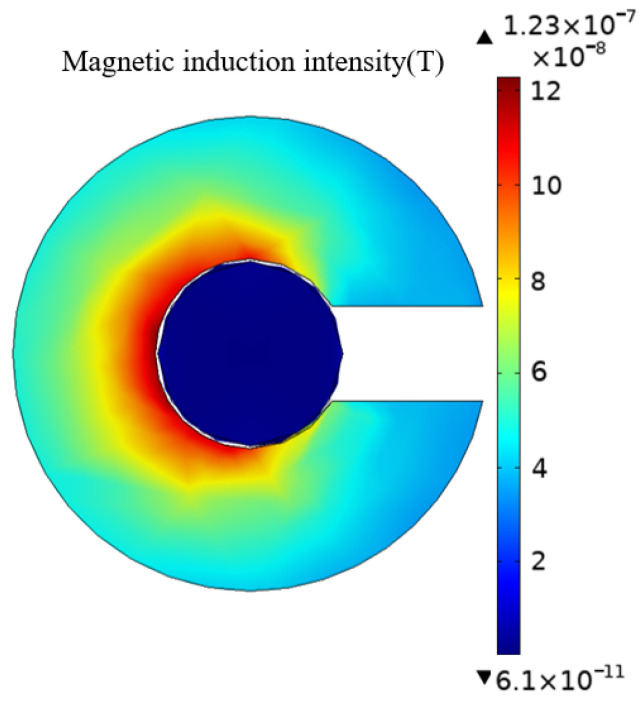
The magnetic induction density of the horizontal plane in which the sensor is located.

Magnetic leakage occurs at the opening of the magnetic ring and reduces the magnetic flux detected by the TMR sensor, so it is critical to design the appropriate magnetic ring structure. First, the magnetic field distribution in the horizontal plane of the sensor is simulated, as shown in Figure 2.

According to theoretical analysis, the closer to the current position, the greater the magnetic induction intensity, and there is magnetic leakage at the edge. As can be seen from the figure, due to magnetic leakage, the magnetic flux density at the air gap of the magnetic concentrating ring is much reduced compared to the inside of the magnetic ring. The magnetic induction density value on the inside of the opening is higher than on the outside. The sensor should be placed in the middle of the air gap for the sake of magnetic field uniformity.

**Figure 3:**
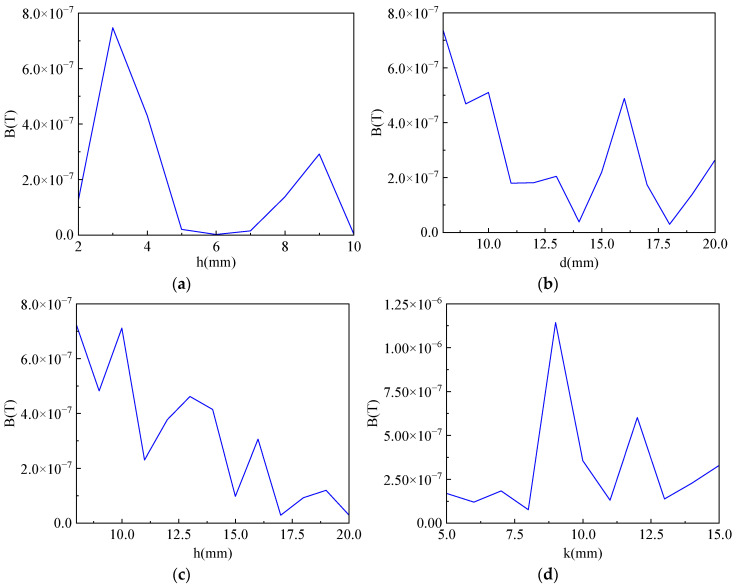
Parametric sweep results of the magnetic concentrating ring size. (**a**) the height; (**b**) the air gap width; (**c**) the inner radius; and (**d**) the thickness.

To explore the influence of the structure size of the magnetic ring on the magnification of the magnetic field at the air gap of the magnetic ring, the height, the air gap width, and the sizes of the inner and outer radii of the magnetic ring were changed, respectively. The change in the outer radius is achieved by changing the thickness of the magnetic ring. Analyzing the changes in the magnetic field at the air gap of the magnetic ring when each variable changed so as to provide some guidance for the determination of the size of the magnetic ring. The size of the magnetic field sensor is 6 mm × 5 mm × 1.5 mm. To ensure that the magnetic field sensor can be wrapped inside the air gap of the magnetic concentrating ring, the height of the magnetic ring cannot be less than 1.5 mm, the air gap width cannot be less than 6 mm, and the thickness cannot be less than 5 mm. Set the range of magnetic ring height h from 2 to 10 mm, the range of magnetic concentrating ring air gap width d from 1 to 10 mm, the inner radius r1 range from 8 to 20 mm, and the ring thickness k range from 5 to 15 mm. The change in magnetic flux density with each parameter is obtained by parametric sweeping, as shown in Figure 3.

When the height of the magnetic ring changes, the magnetic flux density at the center of the air gap fluctuates, and the value of the average magnetic induction intensity detected first increases slightly and then begins to gradually decrease. This is because the magnetic field sensor is always 1 mm from the bottom of the magnetic ring, and the opening edge of the magnetic ring will have some magnetic leakage. When the height of the magnetic ring increases, the magnetic field sensor is farther away from the opening edge of the magnetic ring, so the average magnetic induction intensity increases slightly. However, as the height of the magnet increases, the opening area and magnetic leakage will gradually increase. Therefore, according to the change curve, the height of the magnetic concentrating ring is set to 3 mm.

It can be seen from the figure that the magnetic induction density at the center of the air gap shows a decreasing trend with the increase of the air gap width, i.e., the smaller the air gap width, the better, consistent with the theoretical analysis results. However, when making a magnetic concentrating ring, it is essential to ensure that the main circuit board of the sensor can be stuck at the air gap. The width of the main circuit board is 6 mm; therefore, the air gap width of the magnetic concentrating ring is set to 8 mm to satisfy the requirements. According to the analysis of magnetic circuit theory, when the inner radius of the magnetic core increases, the magnetic circuit of the magnetic ring will increase, resulting in a decrease in the sensitivity of the sensor. With the increase in the inner radius, the magnetic induction intensity value at the center of the magnetic field sensor shows a decreasing trend. The smaller the inner radius, the better the magnetic concentration effect and the greater the magnetic induction intensity generated at the opening, so the inner radius is set at 8 mm.

The difference between the outer and inner diameters of the magnetic ring is the thickness of the ring. To determine the outer radius of the core, it is necessary to analyze the effect of the thickness of the ring on the magnetic induction strength at the opening. It can be seen that as the thickness of the magnetic ring increases, the average magnetic induction intensity first increases slightly and then begins to gradually decrease. This is because the magnetic field sensor as a whole is located in the center of the magnetic ring section, and because there is less magnetic leakage at the center, the detected magnetic field strength will increase somewhat. As the thickness of the magnetic ring continues to increase, the opening size of the magnetic ring increases, and the length of the magnetic circuit of the magnetic ring also increases, which will lead to more magnetic leakage and a decrease in the detected magnetic induction intensity. The increase in ring thickness affects the increase in magnetic induction intensity detected by the magnetic field sensor, and the maximum value is reached at a thickness of 9 mm, i.e., an outer radius of 17 mm is optimal.

In summary, this paper determines that the size of the TMR current sensor’s magnetic concentrating ring is 3 mm in height, 8 mm in air gap width, 8 mm in inner radius, and 17 mm in outer radius. The purpose of improving the sensitivity of the sensor is achieved by optimizing the structure and size of the magnetic concentrating ring.

## 3. Analysis of Electromagnetic Characteristics of the Arrester Simulation Model

### 3.1. Establishment of Arrester Simulation Model

At present, the research on the leakage current of the arrester is mainly focused on field experiments, and almost no research has analyzed the field source distribution of the arrester leakage current detection scene. To show the leakage current of the arrester and its magnetic field distribution, first, you need to obtain the field distribution in its normal state and guide the sensor deployment according to the magnetic field distribution characteristics. Meanwhile, the simulation results of the normal situation can also be used as a control group to analyze the change in the field distribution of the defect.

**Figure 4:**
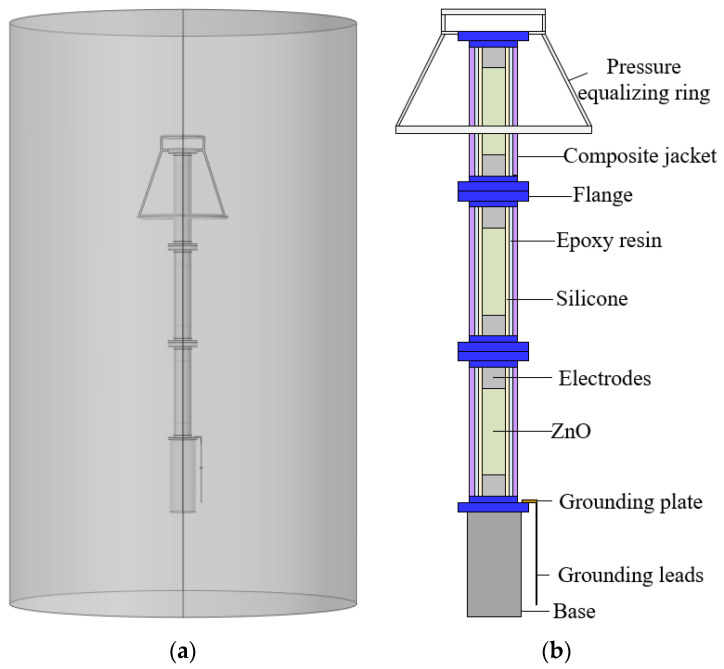
The structure of the 500 kV ZnO arrester. (**a**) an overall diagram of the 3D model; (**b**) the structural composition of the arrester.

Taking the 500 kV ZnO arrester as an example, the arrester is composed of a pressure-equalizing ring, a base, and three arrester units of the same height connected in series. The lowest flange and base are directly connected to the earth, and the leakage current of the arrester flows through the external grounding flat iron. According to the structural dimensions and parameters in Table 1, establish a complete three-dimensional structure model of the arrester, as shown in Figure 4.

**Figure 5:**
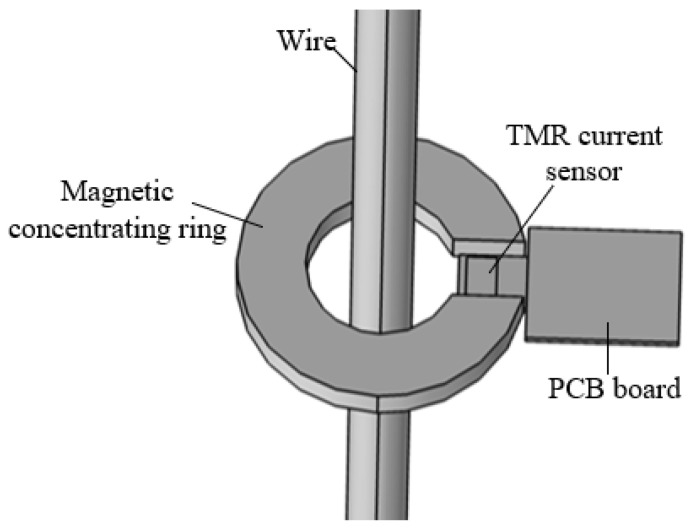
Leakage current measurement.

The model of the TMR current sensor is shown in Figure 5.

Assign values to the relative permittivity, conductivity, and magnetic permeability of each component of the arrester. Boundary conditions are applied to the three-dimensional model, and the single-phase withstand voltage of the 500 kV arrester is 500/$\sqrt{3}$ kV ≈ 289 kV, so the continuous operating voltage of the arrester is 289 kV for the uppermost flange, and zero potential is given to the lowest flange and base. The outside of the arrester is surrounded by air, and all internal boundaries are set to continuous. The boundary condition settings for the model are shown in Table 2.

After the finite element simulation solution is obtained, the electric field, current density, and magnetic field distribution on the arrester are obtained, and the leakage current is calculated by the area fraction of the current density.

**Figure 6:**
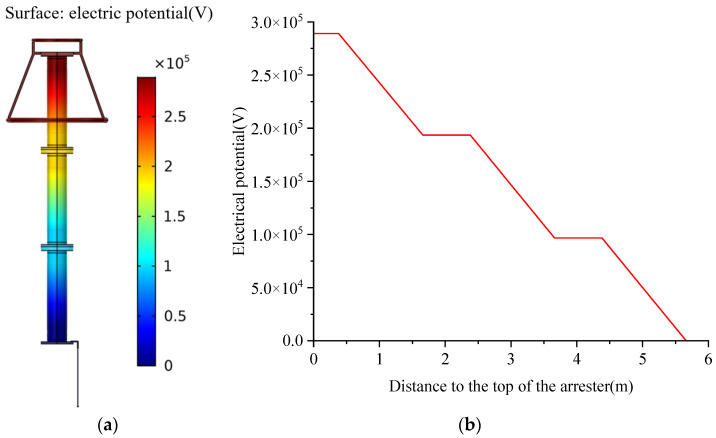
Potential distribution of arresters under normal conditions. (**a**) potential distribution of the arrester; (**b**) potential curve of the arrester.

The potential distribution of the arrester is shown in Figure 6. It can be seen from the figure that the potential distribution of the arrester is in line with reality. The central axis of the arrester is standard, and the potential distribution on the left and right sides is symmetrical. The electric potential at the top of the arrester and the equalizing ring is the highest, and the potential of the rest of the parts gradually decreases from top to bottom. Through the analysis, it can be seen that the adjacent two arresters are connected by a flange, so the potential at the connection is the same. The voltage borne by the upper, middle, and lower arresters is the same; the voltage borne by the upper section is about 95.5 kV, the middle saving is 96.8 kV, and the lower saving is 96.7 kV, and the overall voltage distribution is relatively uniform.

**Figure 7:**
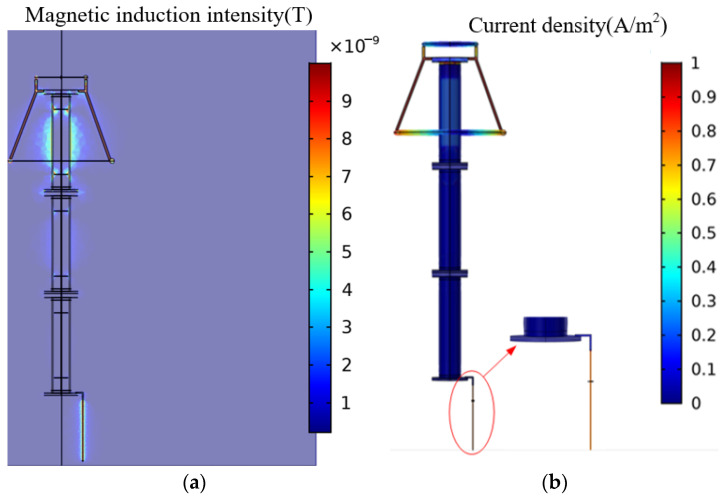
Electromagnetic characteristics of arresters. (**a**) magnetic field distribution; (**b**) current density distribution.

Figure 7 shows the magnetic field and current density distribution of the arrester. It can be seen that when the arrester is in a normal state, the magnetic induction intensity value of the arrester is very small, and the value at the boundary between the arrester electrode and the ground lead is higher than that at other places. The distribution of current density is consistent with the magnetic field distribution.

**Figure 8:**
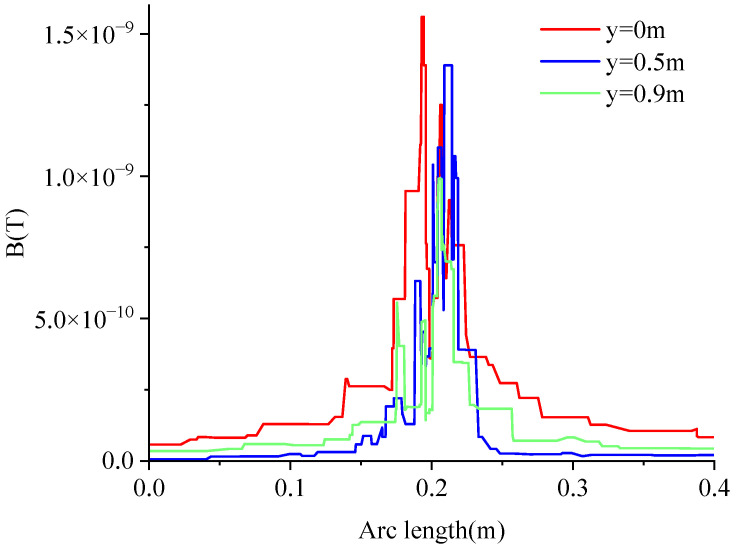
Variation curve of the magnetic field around the ground wire.

The magnetic field around the ground wire is not evenly distributed, and the magnetic field on both sides is not symmetrically distributed. Make a cylinder with a radius of 0.2 m with the grounding wire as the axis, and make a horizontal straight line of 0 m, 0.5 m, and 0.9 m from the bottom end of the ground in the cylinder area to obtain the change of the magnetic field on the straight line, as shown in Figure 8.

It can be seen that the magnetic field is relatively concentrated in the circular area of 0.02 m, the magnetic field in the area outside of 0.02 m decreases sharply, and the maximum magnetic field is relatively larger when it is closer to the bottom end of the arrester, so the TMR sensor chip should be installed in this area as much as possible, and it should also be installed as close as possible to the bottom end of the arrester, which can provide some guidance for the deployment of sensors.

**Figure 9:**
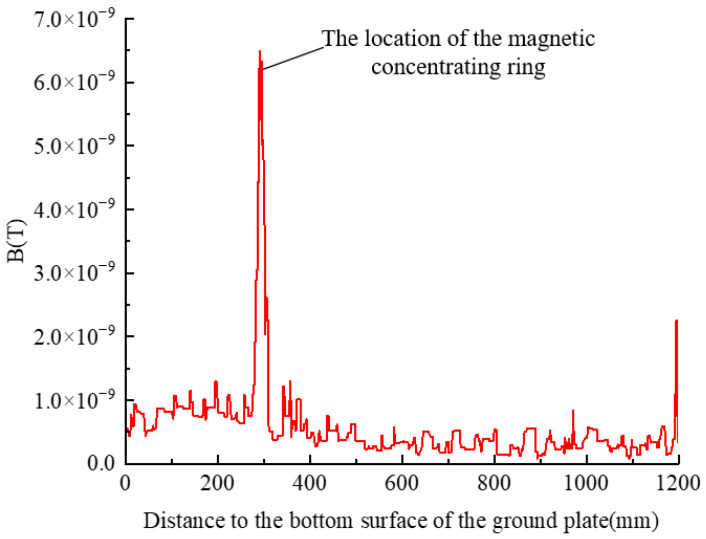
Magnetic flux density changes the curve through the air gap of the magnetic concentrating ring.

The open magnetic ring can amplify the magnetic field at the air gap, and to study the magnetic field amplification effect, draw a straight line through the air gap of the magnetic ring. Figure 9 shows the change in magnetic induction intensity in a straight line.

It can be seen that the magnetic flux density at the non-magnetic ring is very small, and the magnetic induction density at the magnetic ring is significantly enhanced, being about 14 times that of the non-magnetic ring. Since the leakage current value generated by the arrester is too small and the use of magnetic rings can effectively increase the value of the measured magnetic field, the addition of magnetic rings is more conducive to the TMR sensor’s ability to measure the current more accurately.

### 3.2. The Effect of AC Voltage Amplitude on the Leakage Current of the Arrester

The characteristics of ZnO will change due to the working voltage during use, so it is necessary to study the influence of AC voltage amplitude on the leakage current of the arrester. By applying the rated operating phase voltage of 289 kV and the rated voltage of the arrester of 444 kV, 1.3 times the rated voltage, and 1.5 times the rated voltage, the electromagnetic characteristics, and leakage current changes of the arrester are analyzed.

**Figure 10:**
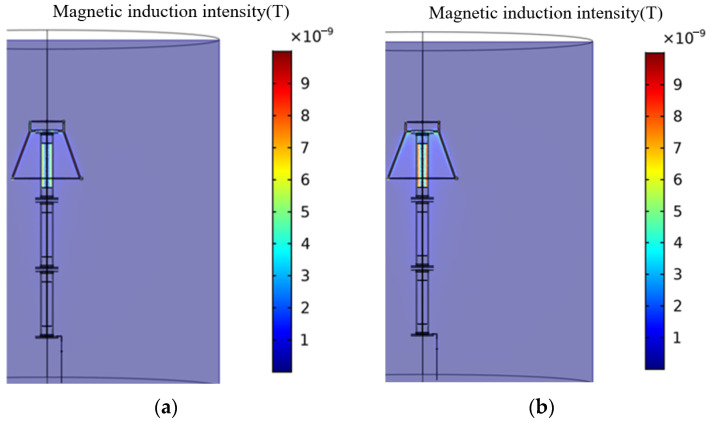

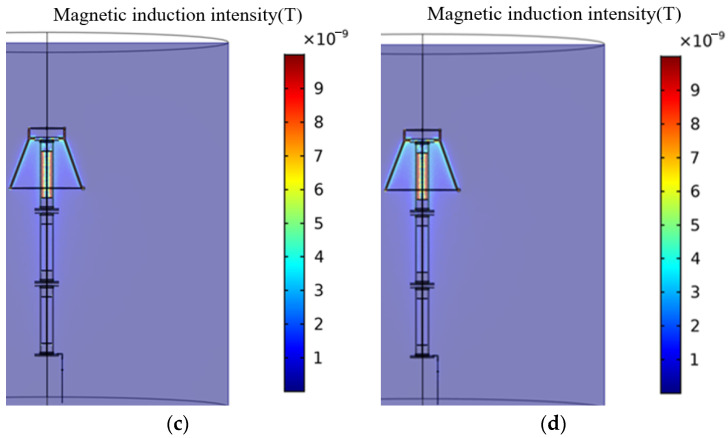
Magnetic field distribution of arrester under different voltages. (**a**) 289 kV; (**b**) 444 kV; (**c**) 577 kV; and (**d**) 666 kV.

The magnetic field distribution obtained by the solution is shown in Figure 10. It can be seen that the magnetic field is mainly concentrated in the upper part of the arrester. When the voltage subjected to the normal arrester gradually increases, the magnetic induction intensity everywhere increases, and the magnetic field aggregation range expands. It can be concluded that the operating voltage of the arrester has a significant impact on the distribution of the surrounding magnetic field.

**Figure 11:**
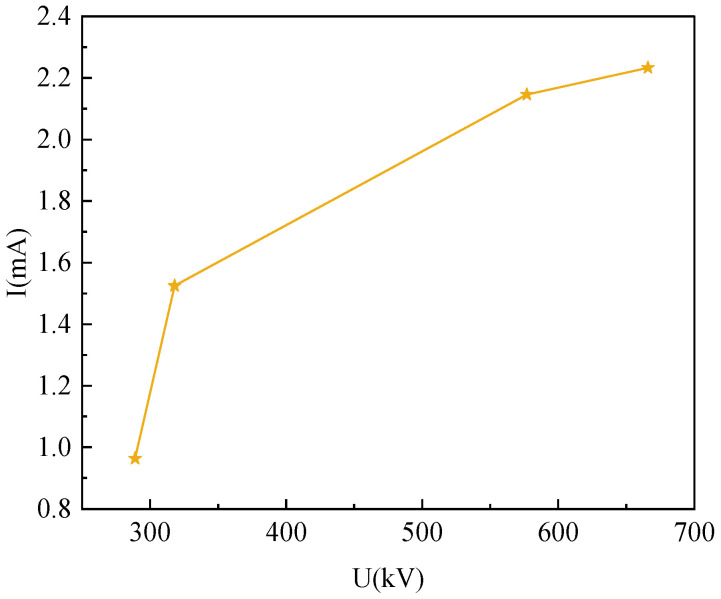
The leakage current changes with the amplitude of the AC voltage.

The leakage current at different voltages is simulated, and the amplitude of the leakage current changes as the applied AC voltage increases, as shown in Figure 11. According to the simulation results, it can be seen that when the AC voltage is applied to 289 kV, 444 kV, 577 kV, and 666 kV, the leakage current is 0.964 mA, 1.53 mA, 2.146 mA, and 2.233 mA, respectively. As the amplitude of the AC voltage applied to the arrester increases, the value of the leakage current increases significantly. When the voltage that the arrester is subjected to increases from the normal operating phase of 289 kV to the rated voltage of 444 kV, the leakage current increases by 58.7% compared to the normal initial value. This indicates that the arrester has undergone overvoltage and serves as a reminder for maintenance personnel to be vigilant about its operating status. When the voltage value increases above 577 kV, the leakage current value becomes more than twice the initial value, indicating that the operating status of the lightning arrester has become abnormal and it should be immediately shut down and repaired. Therefore, the overvoltage operation can be detected in time by monitoring the change in the amplitude of the leakage current.

### 3.3. Electromagnetic Characteristics of Arresters under Moisture

**Figure 12:**
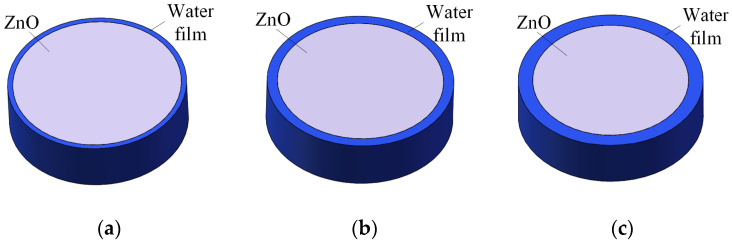
Water film model. (**a**) slight wetness; (**b**) moderate wetness; and (**c**) severe wetness.

Moisture is an important cause of abnormal leakage currents in the arrester, but the specific influence of moisture on the internal insulation performance of the arrester needs further research. This section simulates different moisture states through finite element simulation, obtains the current and electromagnetic field distribution of the arrester, and analyzes the influence of moisture on leakage current more intuitively. For the moisture exposure of ZnO valve plates, water film models of different thicknesses are applied to simulate moisture, as shown in Figure 12. The thicker the water film, the deeper the degree of moisture; the conductivity of the water film is 0.02 S/m, the relative permittivity is 81, and the relative permeability is 1.

**Figure 13:**
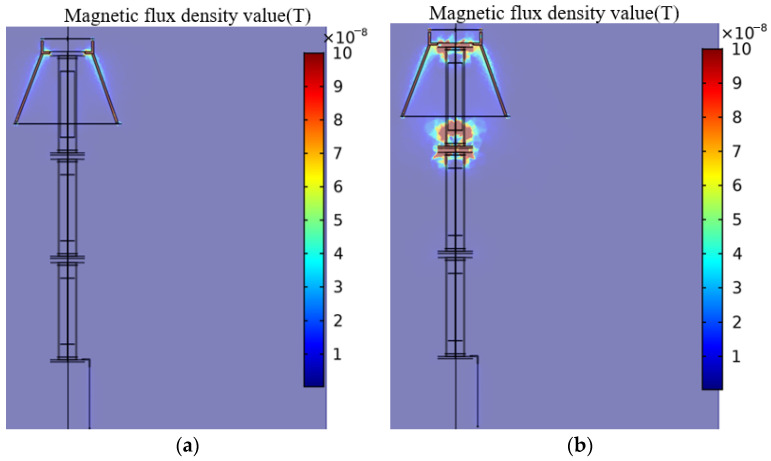

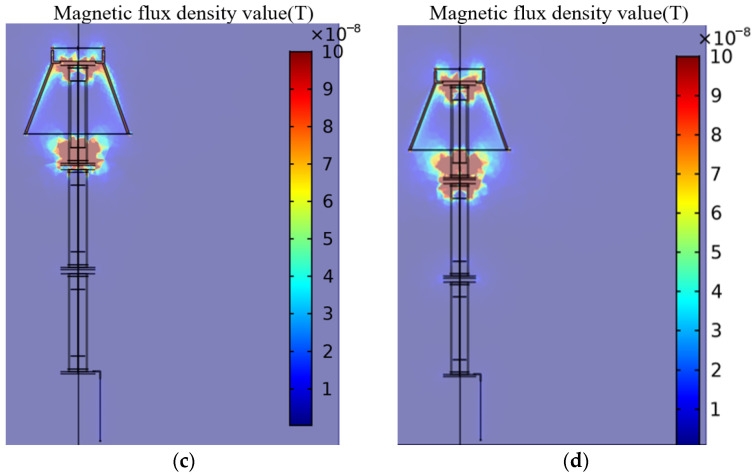
Magnetic field distribution at different degrees of moisture. (**a**) dry condition; (**b**) slight wetness; (**c**) moderate wetness; and (**d**) severe wetness.

The arrester is simulated under 289 kVrms conditions, and the magnetic field distribution is shown in Figure 13.

It can be seen from the comparison of magnetic field strengths that the magnetic field distribution is very weak when it is not moist. With the deepening of the moisture degree of the zinc oxide valve plate, the range of the magnetic field concentration area has expanded. That is due to the presence of a thicker water film on the surface of the valve plate, which increases conductivity along the surface of the valve plate and provides an easier path for the current, so the magnetic field distribution range is also expanded.

**Figure 14:**
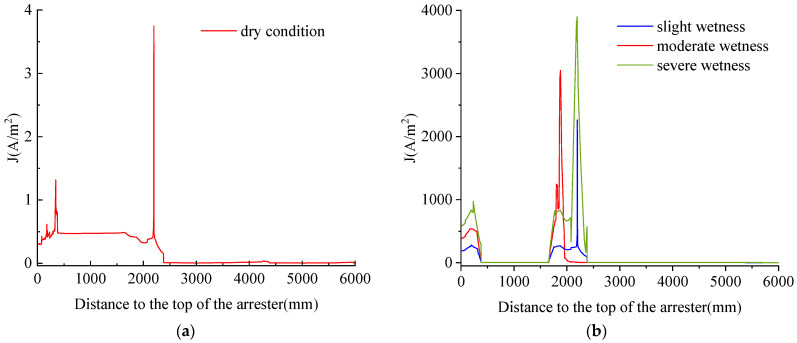
Changes in the current density of the arrester under different moisture conditions. (**a**) dry conditions; (**b**) different degrees of moisture.

To more intuitively compare the influence of moisture on the leakage current, a line segment is made from the top flange of the arrester to the center of the bottom one, and the comparison of the current density on the line segment under the normal condition and different degrees of moisture is obtained, as shown in Figure 14.

**Figure 15:**
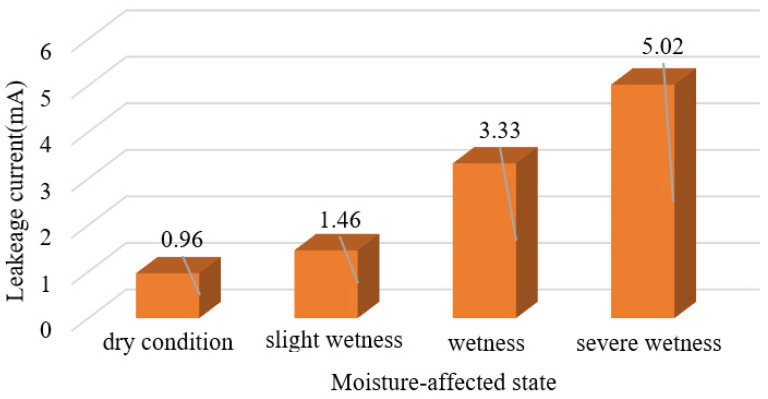
Changes in leakage current under different moisture conditions.

Comparing the changes in current density in the straight line under normal and moisture conditions, it can be seen that the current density is very small when not damp. However, the current density value increases significantly after moisture, and as the degree of moisture increases, the current density also increases. The change of leakage current when the arrester valve plate is not exposed to moisture and when the degree of moisture gradually increases, as shown in Figure 15.

Observing the above figure, it can be seen that the leakage current is 0.96 mA when not damp, and the leakage current increases to 1.46 mA when it is slightly damp. Although the increase in amplitude is small, it also indicates that the state of the surge arrester has been affected to some extent, and if not treated, its lifespan may be shortened. With more serious dampness, the leakage current increases nonlinearly, and when it is moderately damp, the leakage current increases by 128% compared with slight moisture. It indicates that the state of the surge arrester has been significantly affected and that its lifespan will be noticeably shortened. And when it is severely damp, the leakage current reaches 5.02 mA, with the leakage current value already abnormal. The lifespan of the surge arrester will be greatly affected, and it needs to be replaced or repaired on time. According to the difference in leakage current in different moisture conditions, in practical applications, the leakage current measured by the current sensor can reverse the state of the internal valve plate of the arrester, which provides certain guidance for the state evaluation of the arrester.

## 4. Leakage Current Measurement Experiment

### 4.1. Leakage Current Experiment Platform

Since the 500 kV arrester is usually installed in the substation and it is difficult to control its moisture degree, the 10 kV arrester is used for the leakage current experiment. Prepare four identical arresters. This experiment includes two groups: the first group is the leakage current test of the undamped arrester at different voltages, and the second group is the leakage current test of the damp arresters. One of the arresters was used to apply different voltages for the test, and the remaining three arresters were treated with moisture. They were soaked in saline solution, and the leakage current after soaking the samples for 5 days, 15 days, and 30 days was measured, and the relationship between the soaking time and the degree of moisture is shown in Table 3.

**Figure 16:**
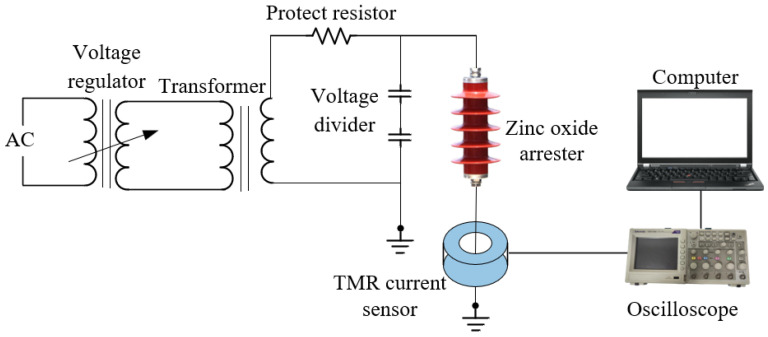
Schematic diagram of the leakage current experiment platform.

**Figure 17:**
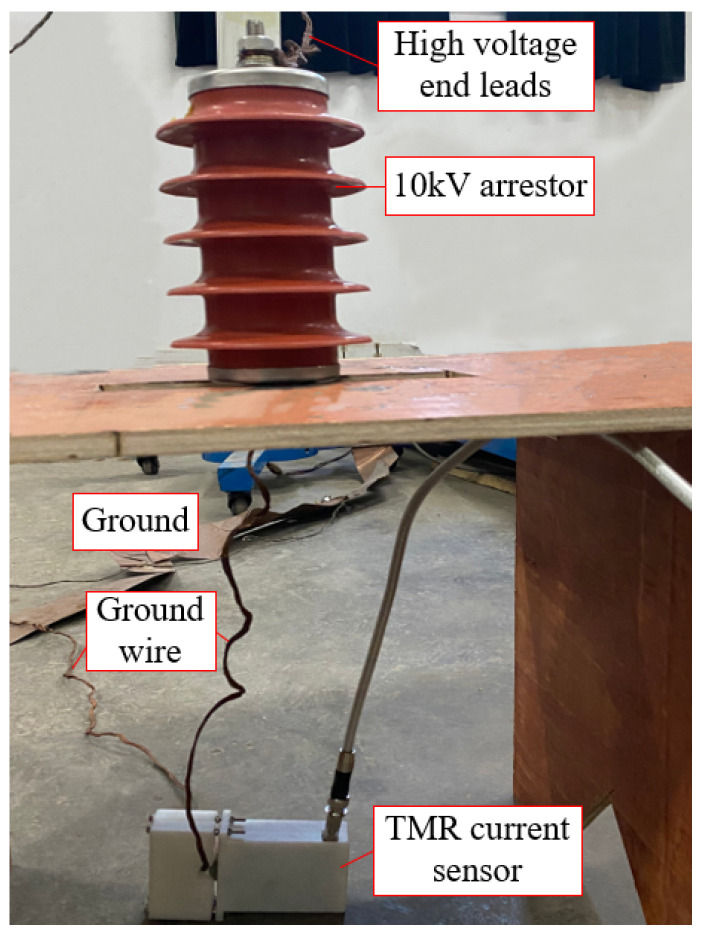
Field experiment diagram of leakage current.

The leakage current measurement test is carried out on the arrester in a normal state and different moisture states, and the connection diagram is shown in Figure 16, and the actual connection diagram in the field is shown in Figure 17.

The main equipment models used in the experiment are shown in Table 4. Among them, the input voltage of the console is 380 V, and the output voltage is 0–400 V. The transformer has a rated input voltage of 380 V, an output voltage of 0–100 kV, and a rated capacity of 30 kVA.

### 4.2. Results and Analysis of the Experiment

#### 4.2.1. Analysis of Leakage Current of Arrester under Different AC Voltages

Measurements of leakage current values at different voltages were performed using the TMR current sensor proposed in this paper and the current clamp meter from Guangzhou Yitai Electronic Technology Co., Ltd (Guangzhou, China). The parameters of the current clamp meter and TMR current sensor are compared in Table 5. The current clamp meter was considered the standard device for measuring leakage current, and the measurement results of the TMR current sensor proposed in this article were compared with it. However, the current clamp meter is only suitable for temporary detection and cannot be installed on the arrester for long-term monitoring. Therefore, if the measurement results of the TMR current sensor proposed in this article are consistent with those of the current clamp meter, it indicates that the proposed current sensor can be used for leakage detection of lightning arresters.

**Figure 18:**
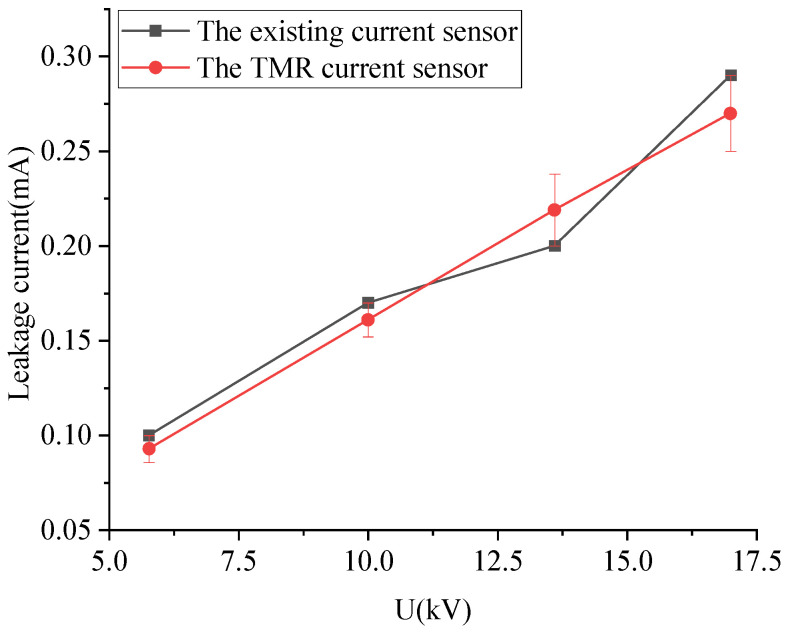
Comparison of leakage current at different voltages measured by the TMR sensor and the existing sensor.

Normal arresters were tested at 5.77 kV, 10 kV, 13.6 kV, and 17 kV, respectively. The measurement results are shown below in Figure 18.

As can be seen from Figure 18, the leakage current measured by the TMR sensor and the current clamp meter is 0.096 mA and 0.1 mA at 5.77 kV, 0.16 mA, and 0.17 mA at 10 kV, 0.21 mA and 0.2 mA at 13.6 kV, and 0.28 mA and 0.29 mA at 17 kV. As the voltage increases, the errors are, respectively, 4.1%, 4.7%, 4.5%, and 3.8%. It can be found that the sensor proposed in this paper can accurately measure the leakage current value, and with the increase in AC voltage, the measured leakage current amplitude also increases, which can verify that the operating voltage value will have a greater impact on the leakage current. When the applied voltage is the rated phase voltage of 5.77 kV, the TMR current sensor measures a current of 0.096 mA. However, when the voltage increases to 10 kV line voltage, the leakage current value does not change significantly, but the current increases by 66.7%, indicating a tendency for overvoltage in the arrester. Therefore, preventive measures should be taken, and inspections should be strengthened. When the voltage rises to the rated voltage of the arrester, which is 17 kV, the leakage current has doubled compared to when the rated phase voltage was applied. This indicates that the overvoltage in the arrester is severe, and long-term operation at this voltage will shorten the life of the lightning arrester. Therefore, equipment inspection should be carried out, and any abnormalities should be promptly shut down.

In addition to current clamps, compared with other arrester monitoring devices, the sensor proposed in this article has high measurement sensitivity and is very suitable for measuring leakage currents. Besides, its advantages, such as its small size, lightweight, flexibility, and convenient installation, are conducive to achieving distributed measurement and facilitating large-scale promotion and application. Thus, the leakage current sensor proposed in this paper is more beneficial for current measurement in practical engineering.

#### 4.2.2. Analysis of Leakage Current of Damp Arrester under Line Rated Phase Voltage

**Figure 19:**
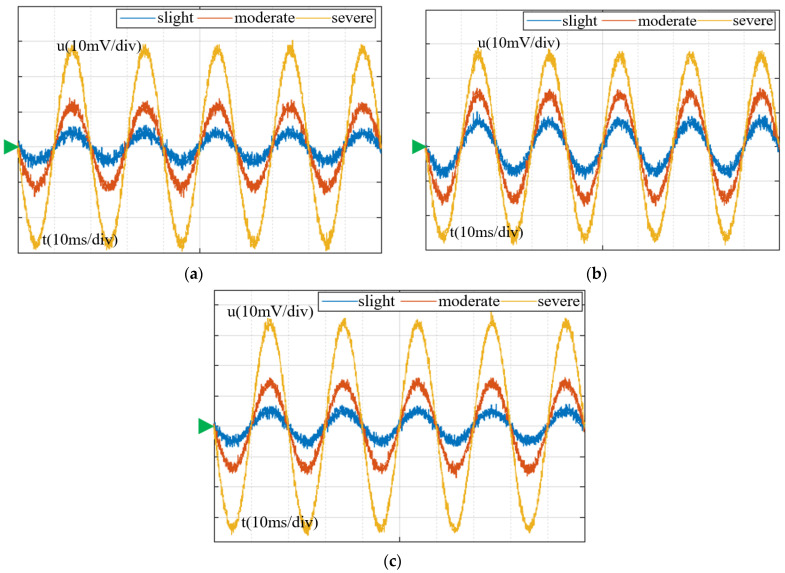
Voltage waveforms change in different moisture states of each sample. (**a**) sample 1; (**b**) sample 2; and (**c**) sample 3.

To be as close as possible to the actual operation of the power system, a rated operating phase voltage of 5.77 kV is applied to the arresters. The test observes the change in leakage current when the arrester is wet. As shown in Figure 19, the voltage waveform measured is carried out after three arrester samples are tested for slight, moderate, and severe moisture. The green triangle in the figure indicates the baseline.

The current measurement is realized indirectly through the TMR sensor, which belongs to the magnetic field sensor. When a conductor is energized, a magnetic field is generated around it. The internal bridge resistance of the TMR sensor changes with the influence of the magnetic field, and the output voltage of the sensor also changes accordingly. That is, the TMR sensor can convert the magnetic field signal into a voltage output. After obtaining the output voltage, the sensitivity of the current sensor is used to determine the magnitude of the measured current.

**Figure 20:**
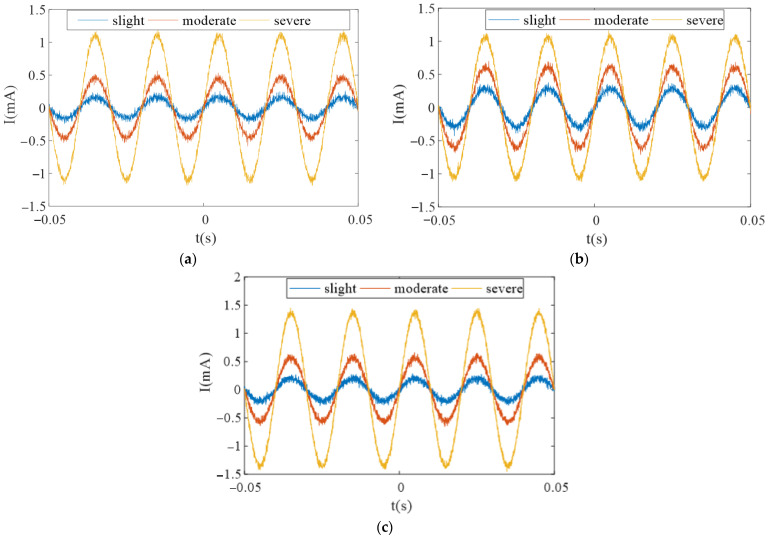
The change of leakage current in the different moisture states of each sample. (**a**) sample 1; (**b**) sample 2; (**c**) sample 3.

Sets of current values are set through experiments, and multiple sets of output voltage values of the TMR sensor are obtained that satisfy a linear relationship. By using the measured voltage-current data to draw a straight line, the slope of the line is calculated to be 25 (mV/mA). According to this value, the voltage waveform is converted to a current waveform, as shown in Figure 20.

It can be seen from the figure that the leakage current of the three arresters rises with the increase in the degree of moisture, which effectively verifies the changing trend of the simulation. The RMS values of leakage current under different conditions were obtained for three sets of surge arresters. Since the values obtained for each set of surge arresters were similar, the average value of the RMS values for the three sets of surge arresters was taken to represent the overall change in leakage current under different degrees of moisture. When slightly damp, the RMS value of the leakage current is approximately 153 μA, while it is 384 μA and 827 μA for moderate and severe moisture, respectively, which is consistent with the simulation.

## 5. Conclusions

In this paper, the leakage current of the arrester under a normal state and different moisture degrees is analyzed by the method of simulation combined with the experiment, and it is observed that the leakage current is significantly affected by the operating voltage and humidity. The design of the magnetic concentrating ring size can improve the detection sensitivity of the TMR current sensor, ensuring that the sensor accurately measures the small leakage current. Through the three-dimensional modeling of the arrester, the distribution of electric potential, current density, and magnetic field of the model is determined, which guides the deployment of the sensor. Through the simulation of different AC voltages and moisture, the magnetic field and current changes generated by the change in insulation performance during the operation of the arrester are more intuitively presented, and the leakage current results under overvoltage and moisture conditions are successfully reproduced, which can be used for the state evaluation of the arrester. The consistency of field trials and simulations verifies the effectiveness of the simulation.

In future work, to ensure that TMR can measure the leakage current more accurately, the interference generated by the surrounding equipment will be considered, the distribution of the leakage current field of the arrester will be studied in depth, and measures to weaken the interference will be proposed in a targeted manner.

## Figures and Tables

**Table 1:** External dimensions of 500 kV arrester.

Model	Height (mm)	Component Height (mm)	Creepage Ratio (mm/kV)	The Outer Diameter of Sheds (mm)	Weight (kg)
Y20W-444/1106	6280	1880	≥31	Φ430	1550

**Table 2:** Boundary condition settings.

Boundary	Boundary Conditions	Expression
Air boundary	Magnetic insulation	***n*** × **A** = 0
Ground	Zero potential	V = 0
Terminal	Operating voltage	V = V_0_ (frequency)
All inner boundaries	Conservation of current	$\begin{array}{c} \nabla \cdot J=Q_{j,v}\\ J=\sigma E+j\omega D+J_{e}\\ E=-\nabla V \end{array}$

**Table 3:** The relationship between the soaking time and the degree of moisture.

Degree of Moisture	Soak Duration (Day)
Slight	5
Moderate	15
Severe	30

**Table 4:** Model of the experimental equipment.

Experimental Equipment	Model
Console	KZT(Z,X)-30/0.5
Transformer	YD1W-30/100
Voltage divider	SGB-50C
10 kV arrester	HY5WR-17/45
Tek oscilloscope	TBS1104
TMR current sensor	TMR2905

**Table 5:** Parameters of the current clamp meter.

Model	Range	Accuracy	Minimum Detectable Current	Size
ETCR6300	0~60 A	±1.5%	0.01 mA	175 mm × 70 × 38 mm
TMR2905	0~150 mA	±1%	0.01 mA	116 × 55 × 36 mm

## Data Availability

The data that supports the findings of this study are available from the corresponding author upon reasonable request.
